# Introduction of nuclear medicine research in Japan

**DOI:** 10.1007/s00259-016-3468-4

**Published:** 2016-08-04

**Authors:** Masayuki Inubushi, Tatsuya Higashi, Ichiei Kuji, Setsu Sakamoto, Manabu Tashiro, Mitsuru Momose

**Affiliations:** 1 Division of Nuclear Medicine, Department of Radiology, Kawasaki Medical School, 577 Matsushima, Kurashiki, Okayama 701-0192 Japan; 2 National Institute of Radiological Sciences, National Institutes of Quantum and Radiological Science and Technology, 4-9-1 Anakawa Inage-ku, Chiba, Chiba 263-8555 Japan; 3 Department of Nuclear Medicine, Saitama Medical University International Medical Center, 1397-1 Yamane, Hidaka-shi, Saitama 350-1298 Japan; 4 PET Center, Dokkyo University School of Medicine, 880 Kitakobayashi, Mibu, Tochigi 321-0293 Japan; 5 Division of Cyclotron Nuclear Medicine, Cyclotron and Radioisotope Center, Tohoku University, 6-3 Aoba Aramaki Aoba-ku, Sendai, Miyagi 980-8578 Japan; 6 Department of Diagnostic Imaging and Nuclear Medicine, Tokyo Women’s Medical University, 8-1 Kawada-cho Shinjuku-ku, Tokyo, 162-8666 Japan

**Keywords:** ^11^C-4′-Thiothymidine (4DST), IgG4-related disease, ^18^F-FRP170, ^18^F-FE-PE2I, Semiconductor SPECT, Spillover correction

## Abstract

There were many interesting presentations of unique studies at the Annual Meeting of the Japanese Society of Nuclear Medicine, although there were fewer attendees from Europe than expected. These presentations included research on diseases that are more frequent in Japan and Asia than in Europe, synthesis of original radiopharmaceuticals, and development of imaging devices and methods with novel ideas especially by Japanese manufacturers. In this review, we introduce recent nuclear medicine research conducted in Japan in the five categories of Oncology, Neurology, Cardiology, Radiopharmaceuticals and Technology. It is our hope that this article will encourage the participation of researchers from all over the world, in particular from Europe, in scientific meetings on nuclear medicine held in Japan.

## Introduction

Every time we attend annual meetings of the European Association of Nuclear Medicine, we are surprised at the large number of attendees from Japan. In contrast, at the Annual Meeting of the Japanese Society of Nuclear Medicine there were fewer attendees from Europe than expected, probably due in part to the language issue. However, there were many interesting presentations of unique studies in Japan. These include research on diseases that are more frequent in Japan and Asia than in Europe, synthesis of original radiopharmaceuticals, and development of imaging devices and methods with novel ideas especially by Japanese manufacturers. In this review, we discuss recent nuclear medicine research conducted in Japan in the five categories of Oncology, Neurology, Cardiology, Radiopharmaceuticals and Technology.

## Oncology

Okasaki et al. conducted a prospective study in an undeveloped medical field: a comparative study of the diagnostic evaluation of multiple myeloma (MM) using three different PET tracers, ^18^F-fluorodeoxyglucose (FDG), an amino acid tracer ^11^C-methionine (MET), and a tumour proliferation tracer ^11^C-4′-thiothymidine (4DST) which has been recently introduced in Japan [1]. The progression of MM from care-not-required premalignant conditions, including monoclonal gammopathy of undetermined significance (MGUS) and smouldering MM (SMM), to care-required MM with end-stage organ damage requires clinical evaluation, but the diagnostic imaging for this purpose has not been established. In addition, MM occurs as the disease spreading type, focal lytic bone lesions (FLL) and diffuse bone marrow lesions (DBML). These are major challenges for imaging modalities, not only for morphological imaging, but also for PET. Okasaki et al. performed two different studies with relatively large numbers of patients: study 1 in FLL (24 patients, 55 lesions) and study 2 in DBML (36 patients, 36 lesions). In both studies, metabolic PET imaging using MET and 4DST showed higher accuracy than FDG, and clearly distinguished MGUS from SMM and MM in DBML. Their methodology (separate evaluation of FLL and DBML) is feasible in clinical use and may be a standard imaging method for PET/CT evaluation in the diagnosis of MM. Their method may also be applicable to PET imaging using ^18^F-fluorothymidine (FLT), a more commonly used PET tracer for evaluation of tumour proliferation. A follow-up study using FLT would be interesting.

IgG4-related disease (IgG4-RD) has been reported more frequently in Japan than in Europe. However, we believe that knowledge of the exact criteria for the diagnosis of this disease is also important in Europe. Tokue et al. retrospectively evaluated FDG PET/CT images in patients with IgG4-RD to determine the involvement of the head and neck glands in this disease [2]. Despite the small number of patients included (17), they evaluated in detail the clinical manifestations, including the chief complaints, serum blood data, and the pattern of extraglandular involvement including the lung, pancreas, kidney, retroperitoneum, and prostate. They found that almost 90 % of patients showed extraglandular involvement and that almost 95 % showed elevated serum IgG4 values. These findings indicate that serum IgG4 examination is important when a multiple organ involvement pattern is observed on FDG PET/CT. IgG4-RD can easily be differentiated from malignant lymphoma by determination of serum IgG4, rather than by lymph node biopsy. In view of its low invasiveness and saving of medical costs, an understanding of the use of FDG PET/CT for the diagnosis of IgG4-RD would be valuable.

## Neurology

Dementia with Lewy bodies (DLB) is the second most common neurodegenerative dementia following Alzheimer’s disease (AD). The hypometabolic regions observed in DLB brains are similar to those in AD brains, although DLB brains also exhibit involvement of the occipital lobe. There is a controversy regarding the correlation between amyloid deposition and the symptom profile, severity and progression, as some patients with DLB can show a pattern of AD-like reduced glucose metabolism without amyloid deposition. Ishii et al. examined regional hypometabolism and amyloid deposits (using Pittsburgh compound B, ^11^C-PiB) in the DLB brain in order to investigate the relationship between the reduced glucose metabolism in the parietotemporal and posterior cingulate and amyloid deposition in the DLB brain and the degree of regional hypometabolism in DLB in relation to that in AD [3]. They found that in the DLB brain regional glucose metabolism is affected both in subjects positive and in those negative for PiB uptake showing a reduction pattern similar to that in AD subjects, and the degree of metabolic reduction in the parietotemporal and occipital cortices is larger than in AD. These findings may suggest that amyloid deposition in DLB brain has no correlation with reduced glucose metabolism, in contrast to the pattern in AD brain.

Regarding Lewy bodies, the use of an innovative ^123^I-metaiodobenzylguanidine (MIBG) scintigraphy method for distinguishing Lewy body-related disease (LDRD) from Parkinson’s syndrome has been reported. A dual time-point imaging technique involving myocardial MIBG scintigraphy at 10 – 30 min after injection (early phase) and 3 – 4 h after injection (late phase) is commonly used to diagnose neurodegenerative diseases. Shiiba et al. proposed new parameters for detecting LDRD in early-phase dynamic images obtained using myocardial MIBG scintigraphy [4]. The early-phase washout rate and area under the time–activity curve from dynamic images showed a diagnostic performance comparable to that of conventional parameters, the heart-to-mediastinum ratio and washout rate. Thus late-phase myocardial MIBG scintigraphy scanning can be omitted for detecting LBRD, and this may reduce the physical and psychological strain on the patients.

## Cardiology

A semiconductor detector system for cardiac imaging has recently been developed with higher sensitivity and higher spatial resolution than conventional SPECT systems [5]. IQ-SPECT (Siemens) is a dedicated myocardial SPECT system, with a novel multifocal collimator, SMARTZOOM, and cardiocentric and 3D iterative SPECT reconstruction, that requires only 6 – 7 min for one scan. Two investigations of IQ-SPECT have recently been reported in Japan [6, 7].

Matsuo et al. investigated in 40 patients with normal perfusion imaging the distribution of ^201^Tl using IQ-SPECT with and without CT attenuation correction (AC) in comparison with that using a conventional protocol with a low-energy high-resolution (LEHR) collimator [6]. All patients underwent a perfusion scan in the supine position after an adenosine stress test and at rest after injection of ^201^Tl using a Symbia T6 scanner equipped with a conventional dual-headed gamma camera system (LEHR collimator) and IQ-SPECT/CT system. One scan took approximately 7 min. The data from the three acquisitions (LEHR collimator, and IQ-SPECT with and without AC) were compared using a semiquantitative 17-segment model and visual scoring using a five-point scale. The quality of IQ-SPECT images without AC was similar to that of conventional imaging using the LEHR collimator. IQ-SPECT with AC provided better detection of the mid-inferior decreased perfusion but lower apical tracer counts compared with conventional imaging using the LEHR collimator.

Takamura et al. investigated prone ^201^Tl imaging without CT-derived AC acquired by IQ-SPECT [7]. The study group comprised 39 patients who underwent ^201^Tl stress imaging with IQ-SPECT. Delayed scan images were analysed by a 17-segment model and visual scoring using a five-point scale. Prone images were compared with supine images with and without CT AC. The quality of the prone images was similar to that of the supine images both with and without AC. Tracer uptake in the apex was significantly greater in the prone images than in the supine images with AC. Prone images showed better attenuation. Fewer artificial defects were observed in the apex in prone images than in supine images with AC.

The two studies show that both CT AC and prone IQ-SPECT images, despite being acquired in only 6 – 7 min, can show better attenuation and correction of inferior artefacts than those acquired with conventional LEHR collimators.

## Radiopharmaceuticals

Hypoxia plays an important role in tumour malignancy by inducing various biological substances, such as hypoxia-inducible factor-1α (HIF-1α), which is a transcription factor for the hypoxic response, and vascular endothelial growth factor which is closely related to angiogenesis. Thus, hypoxia is one of the most active research areas in the tumour molecular imaging field in Japan. Beppu et al. investigated the correlation between the hypoxic area determined by ^18^F-FRP170 PET and HIF-1α and Ki-67 expression areas determined by immunohistochemical staining in surgical specimens of glioblastoma [8]. The proliferative activity within tumour tissues was assessed and showed high accumulation of FRP170 using immunohistochemical staining for HIF-1α as a marker of cell hypoxia, and Ki-67 as a marker of cell proliferation. High uptake was positively correlated with the percentage of HIF-1α-positive cells, but high uptake does not necessarily suggest low proliferation potential, although proliferation is generally considered to be inversely correlated with hypoxia.

As ^123^I-FP-CIT has been widely used in the past 2 years, dopamine transporter (DAT) imaging in Parkinson’s disease and DLB has become a common research focus in Japan. A simple and useful semiquantitative parameter is necessary in the daily clinical setting. In the case of DAT SPECT, the specific binding ratio as calculated by the Tossici-Bolt method is widely used, although there are several quantitation-related issues associated with brain atrophy and the SPECT reconstruction method. Ikoma et al. investigated the analytical parameters for ^18^F-FE-PE2I, a new PET tracer for DAT, using a combination of simulation and clinical studies [9]. They found that FE-PE2I shows high affinity and good selectivity for DAT. They investigated the feasibility of a semiquantitative method with SUVR as an index of DAT binding in PET studies with FE-PE2I in humans, using a computer simulation procedure and data from normal volunteers. They reported that, in addition to the simplified reference tissue mode (SRTM), BP_ND_ estimated from the SUVR of the target and reference regions using frames of late time points also provided stable estimates and showed good correlations with those obtained by the conventional two-tissue compartment model, even though they were greatly overestimated. Furthermore, they concluded that, for estimation of transporter occupancy, SRTM with 90-min data has better reliability than the SUVR method; however, the SUVR method using late time frames also has the potential to provide transporter occupancy with a short scan length.

## Technology

It is our impression that Japanese manufacturers have gradually regained their liveliness in the development of nuclear medicine imaging devices. Hitachi Ltd (Japan) has recently developed a high-sensitivity brain SPECT system using a cadmium telluride (CdTe) semiconductor detector and a four-pixel matched collimator [10]. To further improve its spatial resolution, they have proposed a new dedicated brain SPECT system in which two opposite detector heads of four are tilted towards the rotation axis to be closer to the brain while the other two opposite detector heads remain parallel to the rotation axis to obtain the complete projection dataset required for image reconstruction without artefacts [11]. Although the idea of tilting the detector heads to be closer to the brain is not new, their unique point is the adoption of parallel-hole collimators for the tilted detector heads, instead of slant-hole collimators as had been proposed. As a result, image resolution is improved not only in the upper but also in central slices of the cerebrum.

Methodological advances are also interesting. ^11^C-Acetate has been used to estimate myocardial oxidative metabolism as well as myocardial blood flow (MBF). It was very important, however, to improve the method for correction of spillover from the left ventricle cavity and left ventricle tissues. Recently, Mori et al. have developed a new method for spillover correction to estimate regional MBF (rMBF) in which two MBF-related parameters derived from ^15^O-H_2_O PET are used [12], instead of the conventional one-parameter method. They initially examined the data from 20 subjects (a pilot group), who underwent both ^11^C-acetate and ^15^O-H_2_O dynamic PET at rest, to determine the relationship between the inflow rate of ^11^C-acetate (*K*1) and MBF from ^15^O-H_2_O based on the Renkin-Crone model. They compared ^11^C-acetate *K*1 with the MBF values derived from ^15^O-H_2_O PET. A validation set comprising the data from an additional 43 subjects was used to calculate rMBFs using the relational expression derived from the pilot-group data. In future, in ^11^C-acetate PET studies, nuclear cardiologists will be strongly encouraged to use this new two-parameter spillover correction method to obtain more accurate and robust MBF values than those obtained from the conventional one-parameter method.

## Conclusion

In this review, we have discussed recent nuclear medicine research conducted in Japan in the five categories of Oncology, Neurology, Cardiology, Radiopharmaceuticals, and Technology. At present, Kyoto, the beautiful historical capital of Japan, stands as a candidate host city for the 2022 Congress of the World Federation of Nuclear Medicine and Biology. If selected, it will be an invaluable opportunity to introduce many more Japanese studies in English, the official language of the Congress. We hope this article will encourage the participation of researchers from all over the world, in particular from Europe, in scientific meetings on nuclear medicine held in Japan.
